# Elastic property of sickle cell anemia and sickle cell trait red blood cells

**DOI:** 10.1117/1.JBO.26.9.096502

**Published:** 2021-09-29

**Authors:** Endris Muhammed, James Cooper, Daniel Devito, Robert Mushi, Maria del Pilar Aguinaga, Daniel Erenso, Horace Crogman

**Affiliations:** a Addis Ababa University, Department of Physics, Addis Ababa, Ethiopia; b Middle Tennessee State University, Department of Physics, Murfreesboro, Tennessee, United States; c Meharry Medical College, Meharry Sickle Cell Center, Department of Internal Medicine, Nashville, Tennessee, United States; d Meharry Medical College, Department of Obstetrics and Gynecology, Nashville, Tennessee, United States; e California State University Dominguez Hills, Department of Physics, Carson, California, United States

**Keywords:** red blood cells, laser trapping, cell mechanics, dielectrophoresis, electrostatic force, potential difference

## Abstract

**Significance:** We introduce a model for better calibration of the trapping force using an equal but oppositely directed drag force acting on a trapped red blood cell (RBC). We demonstrate this approach by studying RBCs’ elastic properties from deidentified sickle cell anemia (SCA) and sickle cell trait (SCT) blood samples.

**Aim:** A laser trapping (LT) force was formulated and analytically calculated in a cylindrical model. Using this trapping force relative percent difference, the maximum (longitudinal) and minimum (transverse) radius rate and stiffness were used to study the elasticity.

**Approach:** The elastic property of SCA and SCT RBCs was analyzed using LT technique with computer controlled piezo-driven stage, in order to trap and stretch the RBCs.

**Results:** For all parameters, the results show that the SCT RBC samples have higher elastic property than the SCA RBCs. The higher rigidity in the SCA cell may be due to the lipid composition of the membrane, which was affected by the cholesterol concentration.

**Conclusions:** By developing a theoretical model for different trapping forces, we have also studied the elasticity of RBCs in SCT (with hemoglobin type HbAS) and in SCA (with hemoglobin type HbSS). The results for the quantities describing the elasticity of the cells consistently showed that the RBCs in the SCT display lower rigidity and higher deformability than the RBCs with SCA.

## 1 Introduction

Several experimental approaches such as rheoscopy, flow channels, ektacytometry, and atomic force microscopy (AFM) have been used to study red blood cell (RBC) deformability, with each technique having its unique strengths and limitations. The typical approach for determining cell morphology is ektacytometry.^1^^–^^4^ It is the primary technique used for measuring RBC deformability by laser diffraction viscometry. However, it provides an average measurement of deformability for the entire RBC population, both the abnormal and normal RBCs, potentially underestimating the abnormal RBC population’s rigidity in the blood.^2^ Like ektacytometry, rheoscopy measures cell deformability as a function of shear force through the microscope.^5^ The mechanical property of a membrane can be determined using micropipette aspiration on lipid vesicles^6^ and AFM on lipid bilayers^7^ or pore-spanning membranes.^8^ Micropipette aspiration experiments conducted on lipid vesicles showed that the lipid degree of saturation and the cholesterol concentration primarily affect the membrane stiffness.^9^^–^^11^ AFM experiments showed that cholesterol and sphingolipids enhance lipid bilayers’ mechanical resistance.^12^^,^^13^ Quantitative phase imaging is an optical technique that has also been used to study the morphology and mechanics of RBCs^13^^–^^15^ and has enabled the development of non-invasive live cell imaging systems.

Another approach is molecular dynamics simulations, which can investigate the effect of lipid content on membrane properties. Molecular simulations have been widely used to elucidate how cholesterol and lipid types influence membrane structure and dynamics.^16^^–^^18^ Both atomistic and coarse-grained simulations have been used to clarify the response of the membrane to the mechanical stress by applying tension to the membrane^19^^–^^21^ or by applying an unsteady deformation to the lipid bilayer.^22^

Since its discovery in the early 1980s by Ashkin,^23^^,^^24^ the laser trapping (LT) technique has become one of the greatest inventions of the 20th century. This novel optical technique has recently been recognized by the Nobel Prize in physics in 2018.^25^ The combination of LT techniques with high-resolution imaging techniques has created a wide range of applications in experimental physics and biomedical research. Some of these applications include transporting a gaseous Bose–Einstein condensate,^26^ elastic properties of the human red blood cells (RBCs)^27^^–^^31^ and cancer cells,^32^ and protein unfolding and uncoiling of DNA strands.^33^^–^^35^

The use of LT techniques in studying the mechanical properties of the human RBCs is primarily motivated by the significance of RBCs’ mechanical properties for its biological functioning and the associated health crisis resulting from the abnormality of these cells, such as sickle cell anemia (SCA) and sickle cell trait (SCT).

After its discovery in the western world by Herrick,^36^ SCA has been identified as a genetic disease in which a point mutation in the β-globin gene located on chromosome 11 has one original nucleotide, adenine, replaced with thymidine. This single nucleotide substitution changes the codon from a GAG to a GTG, resulting in the substitution of the amino acid valine for glutamic acid at the sixth position of the β-globin chain producing the sickle hemoglobin (HbS).^37^^,^^38^ This genetic mutation in the heterozygote state results in SCT, where the RBCs will have normal hemoglobin A (HbA) and abnormal HbS. The homozygote state of the $\beta^{S}$-globin gene mutation results in SCA, a type of sickle cell disease, where the RBCs have HbS as the main hemoglobin type. In SCA, the usually round RBCs change their shape and deformability under deoxygenating conditions, which occlude the blood vessels in the microcirculation.^39^ As a result, patients with SCA suffer from chronic hemolytic anemia, and acute painful vaso-occlusive crises that could lead to a central nervous system vasculopathy causing impaired intellectual development and, in some patients, devastating strokes.^40^^,^^41^ Acute vaso-occlusive episodes cause tissue ischemia and excruciating pain, whereas the resulting chronic multi-organ damage leads to disability and early death.^42^

In SCT, HbS is in a concentration of 35% to 45% inside the RBCs. SCT or carriers are considered healthy and have normal life spans.^43^ However, under certain rare conditions they may develop complications. SCA patients present a myriad of health problems and shortened life span.^44^ Although using the LT technique to measure single RBCs’ deformability is advantageous, it does suffer one major drawback due to the uncertainty in force calibration.^45^^,^^46^ This paper introduces a model for better calibration of the trapping force using an equal but oppositely directed drag force acting on a trapped RBC. We demonstrate this new approach by studying RBCs’ elastic properties from deidentified SCA and SCT blood samples.

## 2 Materials and Methods

In this section, we discuss the two essential components to the study’s material and methods, which include the blood samples and the LT technique used in this study.

### 2.1 Laser Trap Setup

**Fig. 1:**
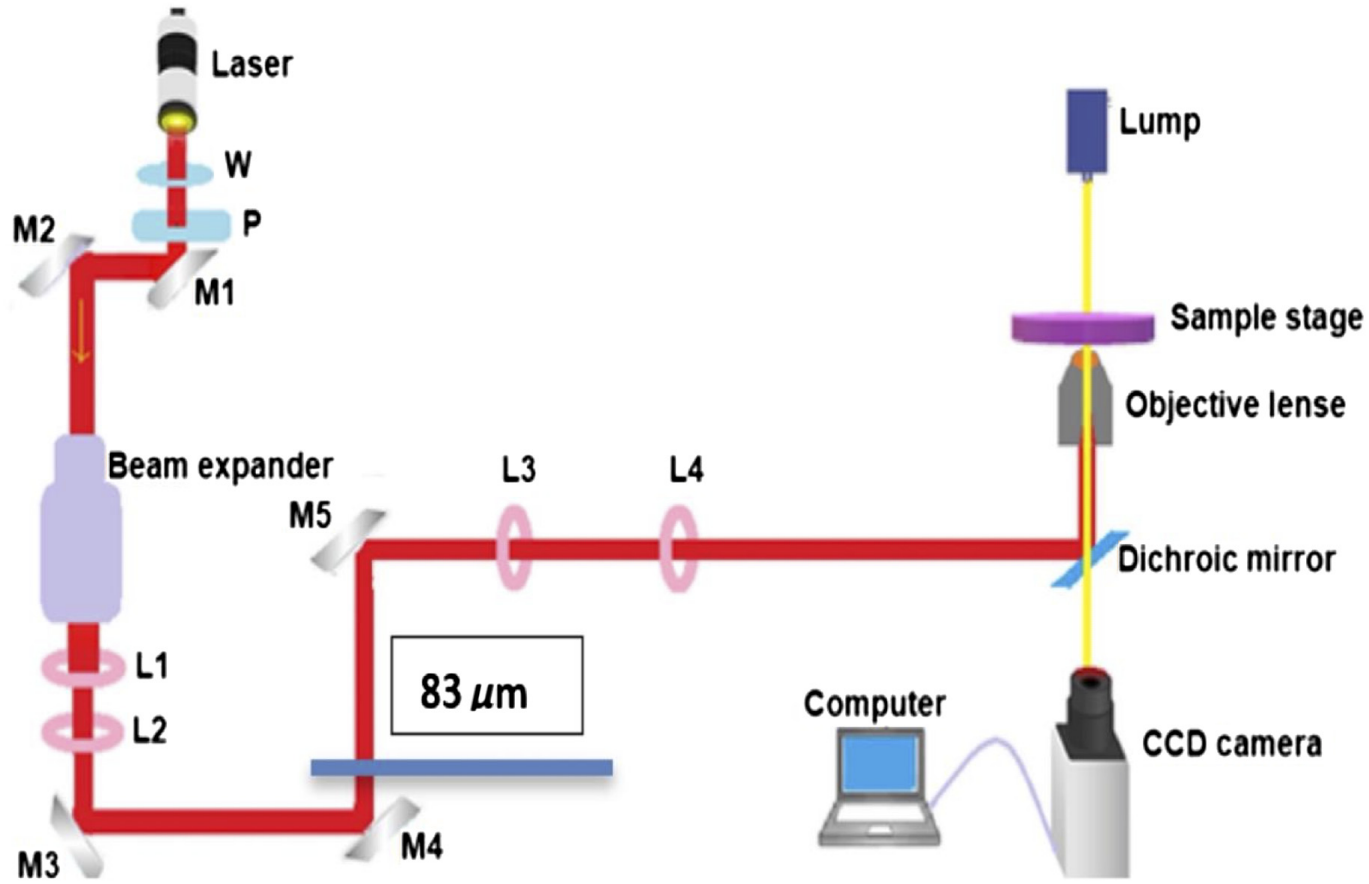
Schematic of the laser trap experimental setup. The laser has a wavelength 1064 nm and maximum power 8 W. The power is controlled by a half-wave plate (W) and a polarizer (P). See Ref. 50 for further detail.

The setup for the laser trap is shown in Fig. 1. This experimental setup is very similar to the setup used in the previous biomedical LT application studies reported.^47^^–^^50^

The laser has a wavelength 1064 nm and maximum power 8 W. The power is controlled by a half-wave plate (W) and a polarizer (P). See Ref. 50 for further detail.

We used an infrared diode-pumped laser lasing at 1064 nm (Spectraphysics V-extreme Nd:YVO4 laser). It generates a linearly polarized beam with a maximum power of 30 mW and beam size of 4 mm. A combination of a half-wave plate (W) and a polarizer (P) was used to control the power of the beam. The beam directed by the mirrors M1 and M2 passes through a 20× beam expander to increase the beam size to about the diameter of the window of the objective lens of the microscope (∼2  cm) as this is critical for a stronger trap. However, since such beam expansion exceeded 2-cm diameter limit, the beam size is readjusted by a pair of lenses L1 and L2 with 5 and 20 cm focal length, respectively. Mirrors M3 and M4 were used for redirecting and better alignment of the beam while M5 was positioned at a proper distance to create steerable trap at the focal plane of the microscope. The position of M5 was 20 cm away from the third converging lens L3 that is positioned from another converging lens L4 (with the same focal lengths of 20 cm) which was placed 20 cm from the back of the objective lens. L3 and L4 were separated by a distance of twice their focal length so that the conditions from geometrical optics are satisfied for the formation of the steerable trap on the focal plane of the microscope. Subsequently, the collimated and aligned beam was coupled to the microscope via a dichroic mirror (DM) position at 45 deg inside the microscope. The DM reflects the laser beam for a normal incidence at 100× objective lens with a 1.25 numerical aperture. At the same time, the DM transmits the imaging light from Olympus halogen lamps for live image captured by a PC controlled digital camera integrated to the microscope via the second port of the microscope.

### 2.2 SCA and SCT Blood Samples Hemoglobin Quantitation and Cell Morphology

In this study, two types of RBC were used: those from SCA and those form SCT blood samples. The deidentified blood samples were obtained from the Meharry Sickle Cell Center (MSCC) of the Meharry Medical College (MMC), Nashville, Tennessee, under a Material Transfer Agreement between MMC and Middle Tennessee State University. The MSCC routinely receives blood samples for hemoglobinopathy diagnosis; for each of these blood samples, the relative percentages of the different Hb types were determined using ultra-high-performance liquid chromatography (UHPLC; Trinity Biotech, Kansas City, Kansas) at the MSCC. The relevant information associated to the two blood samples, such as UHPLC Hb type quantitation and the draw, delivery, and measurement dates for the samples, are tabulated in Table 1. In SCT, the relative percentage of HbS is 33%, whereas in SCA is 97%.

**Table 1:** Basic statistical results for the sizes along with Hb type quantitation measurement by HPLC.

Blood sample		AS	SS
Sex		F	F
Age		20	29
Draw date (M/D/12)		05/17	05/21
Delivery date (M/D/12)		05/23	05/22
HPLC run date (M/D/12)		05/23	05/23
Relative % of each Hb type	HbA (%)	60.00	0.00
	${\mathrm{HbA}}_{2}$ (%)	04.50	02.80
	HbS (%)	32.80	97.20
	HbF (%)	02.70	00.00
Size measurement date		05/25	05/25
Average diameter (μm)		04.02	06.60
Standard deviation (μm)		00.60	00.90

**Fig. 2:**
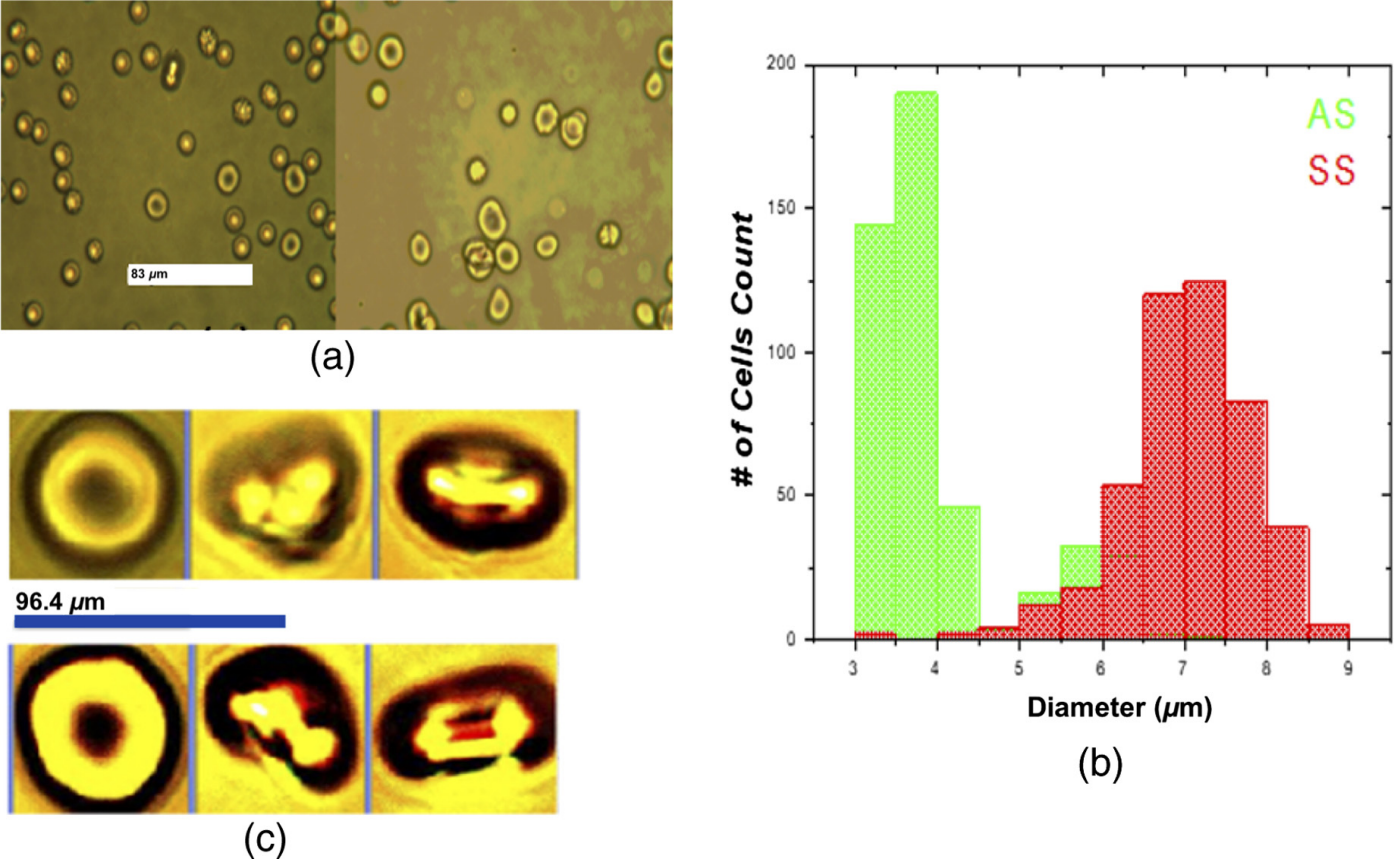
Laser tweezer experiment for comparing the SCT and SCA RBC samples in free and trapped regime. (a) Diluted blood samples SCT (left) and SCA (right). (b) An RBC from an SCT (AS) individual (top three frames) and from an SCA (SS) patient (bottom three frames) taken by, respectively, a free regime, trapped at ∼30  mW, and trapped and dragged RBC at 45  μm/s. (c) The size distribution of the SCT, SCA, and normal blood.

After we received these blood samples, to study HbSS and HbAS RBCs’ elastic property, blood samples were taken and diluted by fetal bovine serum in the ratio 1:1000. For these cells, statistical analyses, based on size measurement, were conducted to study the difference in morphology of the RBCs in the two samples. In Fig. 2(a), the free RBCs for the SCT (left) and SCA (right) is displayed. Using Image Pro Plus 6 image analysis software, we measured the diameter of each cells.^32^ The statistical distribution of these cells is displayed by the Histogram in Fig. 2(b). The mean diameter and the corresponding standard deviation are tabulated in Table 1.

For the elasticity study, the diluted blood from each sample then placed onto a glass slide and covered with a coverslip. The slide was then mounted onto the piezo-driven stage (PS) of the microscope. Free cell snapshots were taken before the sample of HbSS and HbAS were trapped. The power of the laser was ∼30  mW at the tip of the objective lens that pulls the cells to the center of the trap. When the laser port to the microscope opens, the cell enters and becomes instantly trapped. The stage was moved with varying velocities which created a drag force on the trapped cell, causing it to stretch. The sample was subjected to different velocities oscillating in different directions, and the magnitudes of the corresponding drag forces experienced by the cell were determined. The charge-coupled device camera captured successive images of the trapped cell. The procedure was repeated for the seven different RBC groups of HbSS and HbAS. Individual cells in the same subgroup were stretched by moving the PS with the same velocity. Figure 2(c) shows an RBC image from the two blood samples when an RBC is free, trapped, and trapped, and dragged. The top three images display these three states for RBC from SCT and the next three are for an RBC from SCA.

### 2.3 Trapping Force

We consider a simplified physical model for the RBCs to determine the trap force, which is proportional to the magnitude of the drag force. We model an RBC as a thin cylinder with radius R and thickness t, where t≪R.

**Fig. 3:**
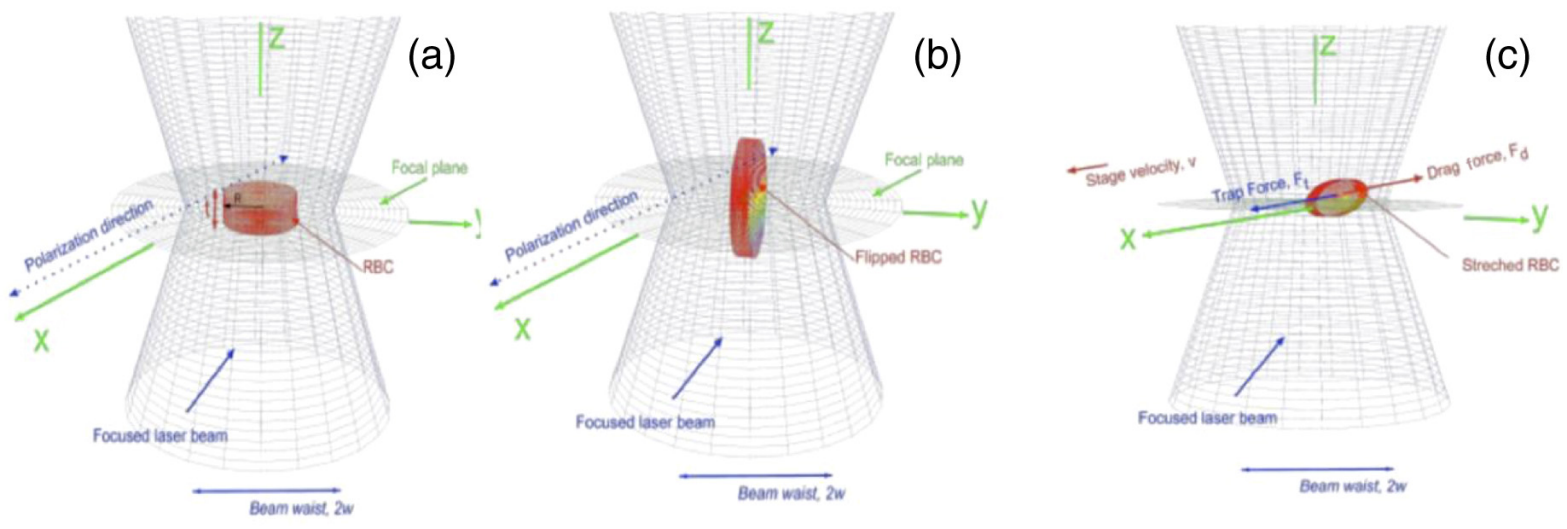
A simplified thin-cylinder model for an RBC: (a) before exposure to the laser beam; (b) when a linearly polarized laser beam propagating along the z direction is turned on and the cell is trapped; and (c) the experience of both trap and drag force as the stage moves with velocity v along the direction of polarization.

The refractive of RBC is higher than water due to the high concentration of hemoglobin molecules. The higher contrast refractive index leads RBC to be electrically polarized under intense high laser beam and easily trapped. In the absence of an external electric field, the dipole moment of these polar molecules is randomly oriented such that the total dipole moment per unit volume, the polarization, $\vec{P}(\vec{r},t)$ is zero. Let us consider an infinitesimal volume $dV^{\prime }$ in the cell as shown in Fig. 5. Let the magnitude of the polarization in this infinitesimal volume be $d\vec{P}$ and the direction ${\hat{r}}^{\prime }$ from the positive z axis. Suppose a laser beam that propagates along the z direction and linearly polarized along the x direction is turned on as shown in Fig. 3. The cell is ionized due to the electric field of the laser $\vec{E}(\vec{r},t)$, it experiences an electrical torque given by

$$\overset{\to }{N}=∭\overset{\to }{P}({\overset{\to }{r}}^{\prime },t)dV^{\prime }\times \overset{\to }{E}({\overset{\to }{r}}^{\prime },t).$$ (1)

Let us assume that there are N molecules in the cell with the magnitude of the dipole moment being p. We can write the net torque on the cell resulting from turning the laser approximately as

N→≃NPEV∫02π∫0R∫−t/2t/2(cos(φ′)x^+sin(φ′)x^+z^)×x^dz′s′ds′ dφ′, (2)

where the volume of the cell is given by $V=\pi R^{2}t$.

The RBC initially sits at the bottom of the slide with its platelet side facing the x−y plane and is forced to rotate about the y axis as a result of the electric torque. However, the cell remains trapped at a position, in which its platelet side facing the x−z plane is parallel to the beam propagation direction as shown in Fig. 3(b). If the stage oscillates with a constant velocity v along the polarization direction, the cell experiences a drag force equal in magnitude but opposite in direction to the trap force shown in Fig. 3(c).

Next, we determined the trap force when the cell’s center mass is moved from the center of the trap and the cell is stretched due to the two opposing drag and trap forces. To this end, we denote the electric field of the laser beam as $\vec{E}(\vec{r},t)$ and the polarization inside the cell as $\overset{\to }{P}(\overset{\to }{r},t)$. The RBC’s center of mass is positioned at $\vec{r}$ measured from the center of the trap as shown in Fig. 3. The trap force can be obtained from

$$\overset{\to }{F}=\int (\overset{\to }{P}(\overset{\to }{r}+{\overset{\to }{r}}^{\prime })\cdot \nabla )\overset{\to }{E}(\overset{\to }{r}+{\overset{\to }{r}}^{\prime })dV^{\prime }.$$ (3)

Due to the electrical toque in the bipolar water molecules, when the cell is trapped, as shown in Fig. 3(b), the axis of the cell becomes normal to the polarization direction of the laser beam. This essentially creates a positive on one side and negative bound surface charges on the other side. This is a physically reasonable approximation comparing the beam radius w of the laser beam at the trap location with the average radius of a human RBC’s R. Since R≪w, we can approximate a uniform electric field over the cell. A closer look at the trapped and dragged cell is shown in Fig. 3.

Suppose the total dipole moment due to the positive and negative bound surface charges on the two opposite sides of the cell is ${\overset{\to }{p}}_{\text{total}}$, the electric field inside the cell can be expressed as

$${\overset{\to }{E}}_{\text{net}}=-\frac{1}{4\pi \varepsilon_{0}}\frac{{\overset{\to }{p}}_{\text{total}}}{R^{3}}.$$ (4)

Using the electric field inside a dielectric cylinder with an electrical permittivity ε placed in a region with electrical permittivity, $\varepsilon_{1}$, and an external electric field $\vec{E}$, we can write the total induced dipole moment as

$${\overset{\to }{p}}_{\text{total}}=-4\pi \varepsilon_{0}R^{3}\frac{\varepsilon -\varepsilon_{1}}{\varepsilon +\varepsilon_{1}}\overset{\to }{E}.$$ (5)

Further, dividing this with the volume of the RBC, $V=\pi \rho^{2}t$ and introducing the refractive indices $n^{2}=\varepsilon /\varepsilon_{0}$ and $n_{1}^{2}=\varepsilon_{1}/\varepsilon_{0}$ for the cell and the medium and also the parameter $m=n/n_{1}$, the polarization can be expressed as

$$\overset{\to }{P}=-\frac{4\varepsilon_{0}R}{t}\frac{m^{2}-1}{m^{2}+1}\overset{\to }{E}.$$ (6)

Now substituting Eq. (5) into Eq. (2), we find

$$\overset{\to }{F}=-\frac{4\varepsilon_{0}R}{t}\frac{m^{2}-1}{m^{2}+1}\int (\overset{\to }{E}\cdot \nabla )\overset{\to }{E}dV.$$ (7)

Applying the identity $\nabla (\overset{\to }{A}\cdot \overset{\to }{B})=\overset{\to }{A}\times (\nabla \times \overset{\to }{B})+\overset{\to }{B}\times (\nabla \times \overset{\to }{A})+\overset{\to }{B}(\overset{\to }{A}\cdot \nabla )+\overset{\to }{A}(\overset{\to }{B}\cdot \nabla )$, one can show that $2\overset{\to }{E}(\overset{\to }{E}\cdot \nabla )=\nabla (\overset{\to }{E}.\overset{\to }{E})-2\overset{\to }{E}\times (\frac{\partial B}{\partial t})$. The second term can be neglected as B∼E/c, where c is the speed of the laser beam in free space. As a result, Eq. (7) becomes

$$\overset{\to }{F}=-\frac{2\varepsilon_{0}R}{t}\frac{m^{2}-1}{m^{2}+1}\int \nabla (E^{2})dV.$$ (8)

Writing the electric field in terms of the intensity of the laser at the trap location by substituting $I=\frac{v\varepsilon_{1}}{2}E^{2}=\frac{c\varepsilon_{1}}{2n_{1}}E^{2}\Rightarrow E^{2}=\frac{2In_{1}}{c\varepsilon_{1}}$ and upon taking the inner product of the force in Eq. (8), the beam propagation direction z^, we may write

F→·z^=−4Rctm2−1m2+1∫∇·(Iz^)dV. (9)

So that using the divergence theorem, ∫∇·(Iz^)dV=∫Iz^·da→ then Eq. (9) can be rewritten as

F→·z^=−4Rctm2−1m2+1∫Iz^·da→, (10)

and there follows that

F→=−4Rctm2−1m2+1∫Ida→=−4Rctm2−1m2+1∫Idas^. (11)

**Fig. 4:**
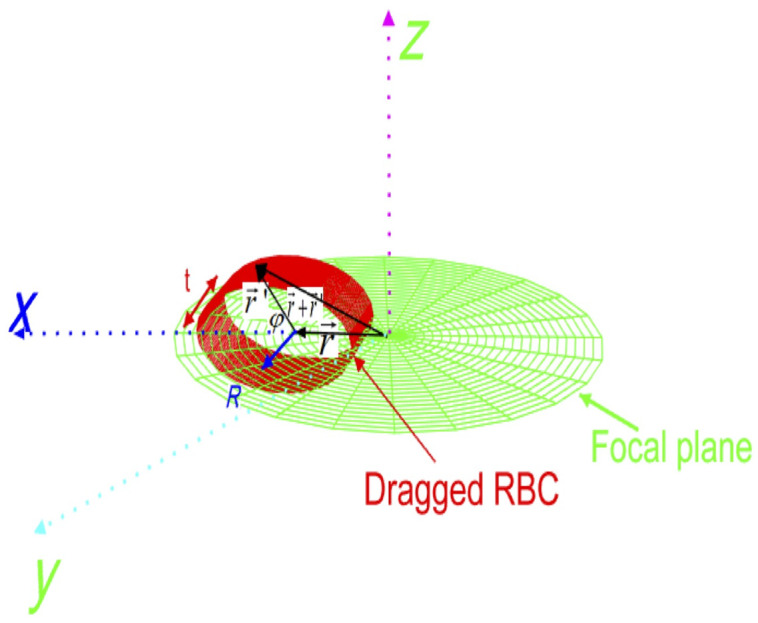
RBC stretched by the dragged force and fixed by trapping force on the other end.

Note that s^ is the unit vector for the infinitesimal area da on the curved part of the cylinder (the RBC). There is no contribution from the platelet sides of the cell as they cancel each other. We now proceed to evaluate the integral in Eq. (10). To this end, it is important to take a closer look at the stretched cell by the drag and trapping forces (see Fig. 4).

The intensity of the Gaussian beam at a point on the curved part of the cylinder can be expressed as

$$I(r)=I_{0}\;\exp[-2({\left(r+R\;\cos\;\varphi \right)}^{2}+R^{2}\;\sin^{2}\;\varphi )/w_{0}^{2}],$$ (12)

where $w_{0}$ is the radius of the beam waist at z=0. Since most of the total power is concentrated within this circle (in the beam waist), then one can write the intensity in terms of the power as

$$I(r)=\frac{2P}{\pi w^{2}}\;\exp[-2({\left(r+R\;\cos\;\varphi \right)}^{2}+R^{2}\;\sin^{2}\;\varphi )/w^{2}].$$ (13)

Noting that for the unit vector normal to the curved part of the cylinder, one can write

s^=x^+cos φx^+sin φz^. (14)

Substituting the infinitesimal area da=Rdφdy, Eqs. (11)–(13) into Eq. (10), one finds

F→=−8PwR2ctπw2(m2−1m2+1)∫02π∫−t/2t/2exp[−2(r2+R2+2R cos φ)/w2]×(x^+cos φx^+sin φz^)dφ dy. (15)

From Eq. (15), the magnitude of the force can be written as

$$F=-\frac{16n_{1}P_{w}\rho^{2}}{cw_{o}^{2}}\frac{m^{2}-1}{m^{2}+1}e^{-\frac{2}{w_{o}^{2}}(\rho^{2}+r_{t}^{2})}J_{1}(0,\frac{4r_{t}\rho }{w_{o}^{2}}).$$ (16)

For the case where $\frac{4r_{t}\rho }{w_{o}^{2}}\ll 1$, the Bessel function $J_{1}(0,\frac{4r_{t}\rho }{w_{o}^{2}})\approx 1$. Under this approximation, the magnitude of the force becomes

$$F=-\frac{16n_{1}P_{w}\rho^{2}}{cw_{o}^{2}}\frac{m^{2}-1}{m^{2}+1}e^{-\frac{2}{w_{o}^{2}}(\rho^{2}+r_{t}^{2})},$$ (17)

where $P_{w}$, ρ, $n_{1}$, m, c, $w_{o}$, and $r_{t}$ are power, the radius of RBC, refractive index of the medium, the ratio of the refractive index of the cell to the medium, minimum spot size, and the distance from the trap to the center of RBCs, respectively. The trapping force in Eq. (17) was used to calculate the numerical data of the trapping force using the measured parameters.

There are a number of approaches in the literature for calculating the trapping force. Ashkin et al.^51^ calculated this force from the Lorentz force acting on the dipole using a spherical model, whereas Bechhoefer and Scott Wilson^52^ determined the optical force from the electromagnetic energy of the particle in spherical model. Susan et al.^53^ and Tlusty et al.^54^ on their end used a maxwell stress tensor formulation to obtain the force. However, our trapping force is derived from the dipole model in cylindrical coordinate using Gaussian beam intensity. This is because a cylindrical model is more appropriate than a spherical model since RBC has a sandwich like structure. All the approaches cited above determined the trapping force analytically using different methods, but all produced similar the results. Conversely, the approach taken in this paper produces a different expression for the polarization due to the cylindrical framework use to model the RBC cell.

### 2.5 Formulation of Free and Stretch Cell Comparison with Trapped SCT and SCA cells

The radius and area of the cells were measured before and after being trapped. The relative change between the trapped cell and the free/stretch cell are used to study the elastic property by measuring the area and radius. We can calculate the relative change between free/stretch and trapped cell size using:

$$\%\mathrm{DR}=\frac{\left(R-R_{T}\right)}{R}\times 100,$$ (18)

where % DR is percentage difference in radius, R represents the radius of the free cell ($R_{f}$) or stretched cell ($R_{S}$), and $R_{T}$ is the radius of the trapped cell. Similarly, we computed change in area for free and stretched cells relative to trapped cells using:

$$\%\mathrm{DA}=\frac{\left(A-A_{T}\right)}{A}\times 100,$$ (19)

where % DA is the percentage difference in area and A can represents the area of the free cell ($A_{f}$) or the area of the stretched cell ($A_{S}$), and $A_{T}$ is the area for the trapped cell. The radius and area for the free, trapped, and stretched HbAS and HbSS RBCs were measured using image analysis software Image Pro plus 6. The resulting data for these percent differences were analyzed using OriginPro data analysis software.

## 3 Results and Discussion

In this section, we will analyze and compare the elasticity property of SCA (HbSS) and SCT (HbAS) RBCs with two different methods. The first method required the trapping of these cells and compressing them. The trapping force is calculated using Eq. (17). The average drag force for each subgroup was 1.48, 1.26, 1.10, 0.853, 0.635, 4.31, and 3.23 pN. The average percent difference in stretched radius and area versus trapped radius and area were calculated.

**Fig. 5:**
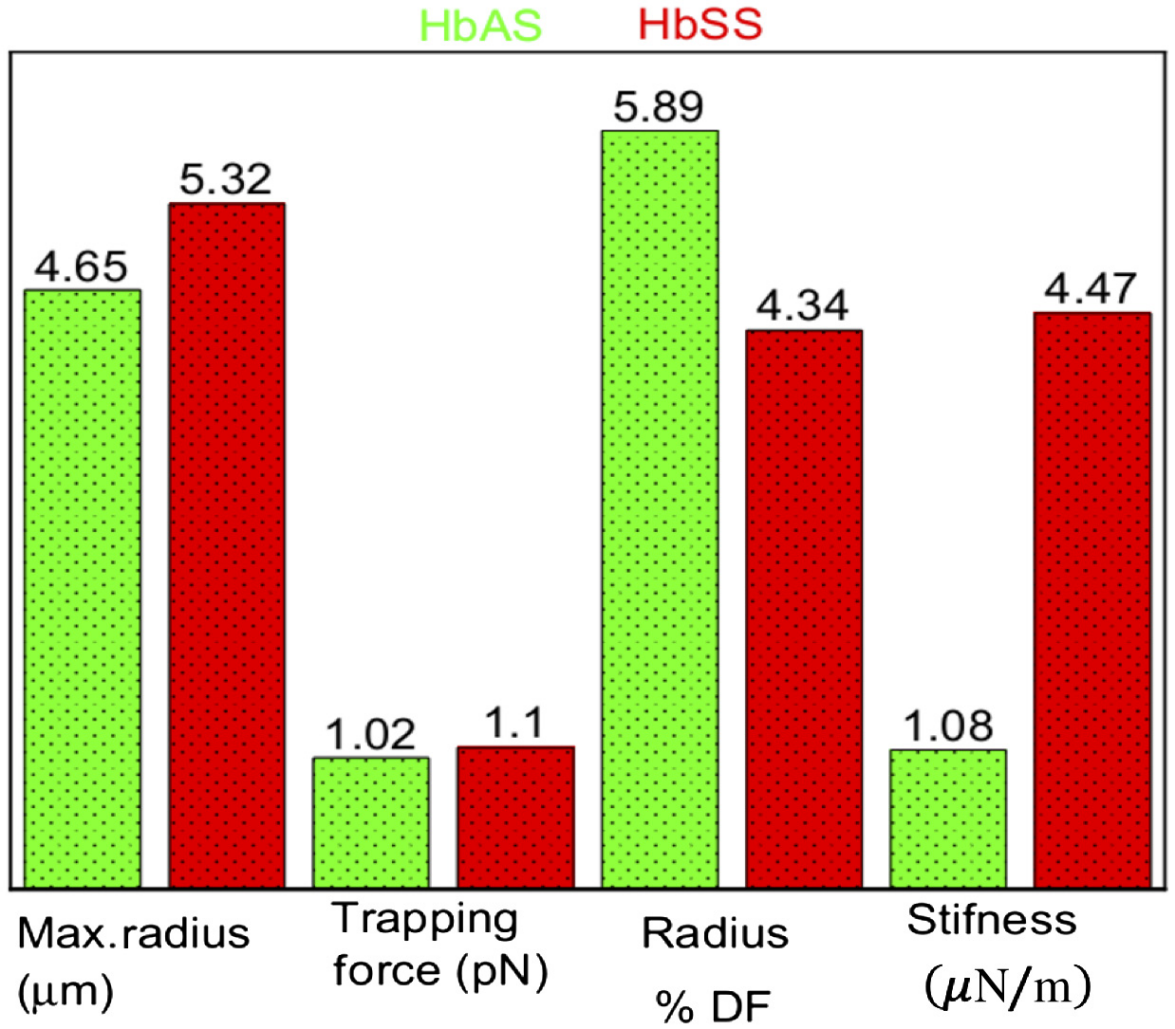
RBC radius, force, radius percent difference, and stiffness are represented by bar graph for the HbAS and HbSS red blood sample.

To conduct this analysis, we used the maximum and minimum radius and area of HbSS and HbAS samples when they are free, trapped, and stretched. The stiffness, relative change of radius, and area of free and stretched cells were calculated relative to the trapped cell. These methods of analysis are presented to show the elasticity property of the HbSS and HbAS. The stiffness k is the change of stretching force over relative change of radius for the individual HbSS and HbAS cells, which is expressed as k=ΔF/ΔR, where ΔR=Rsheer−Rtrap. The radius of HbAS ranges from 3.84 to 5.38  μm with an average of 4.67  μm, which was less than the average of HbSS 5.24  μm that ranges from 4.45 to 6.12  μm. The mean value of the trapping forces are 0.26 and 0.33 pN for the HbAS and HbSS, respectively. The drag forces for HbAS and for HbSS cells are 1.20 and 1.27 pN, respectively. HbSS is stretched with higher force but it has lower relative percent difference than HbAS as shown in Fig. 5.

### 3.1 Free Compared Trapped for SCT and SCA Cells

**Fig. 6:**
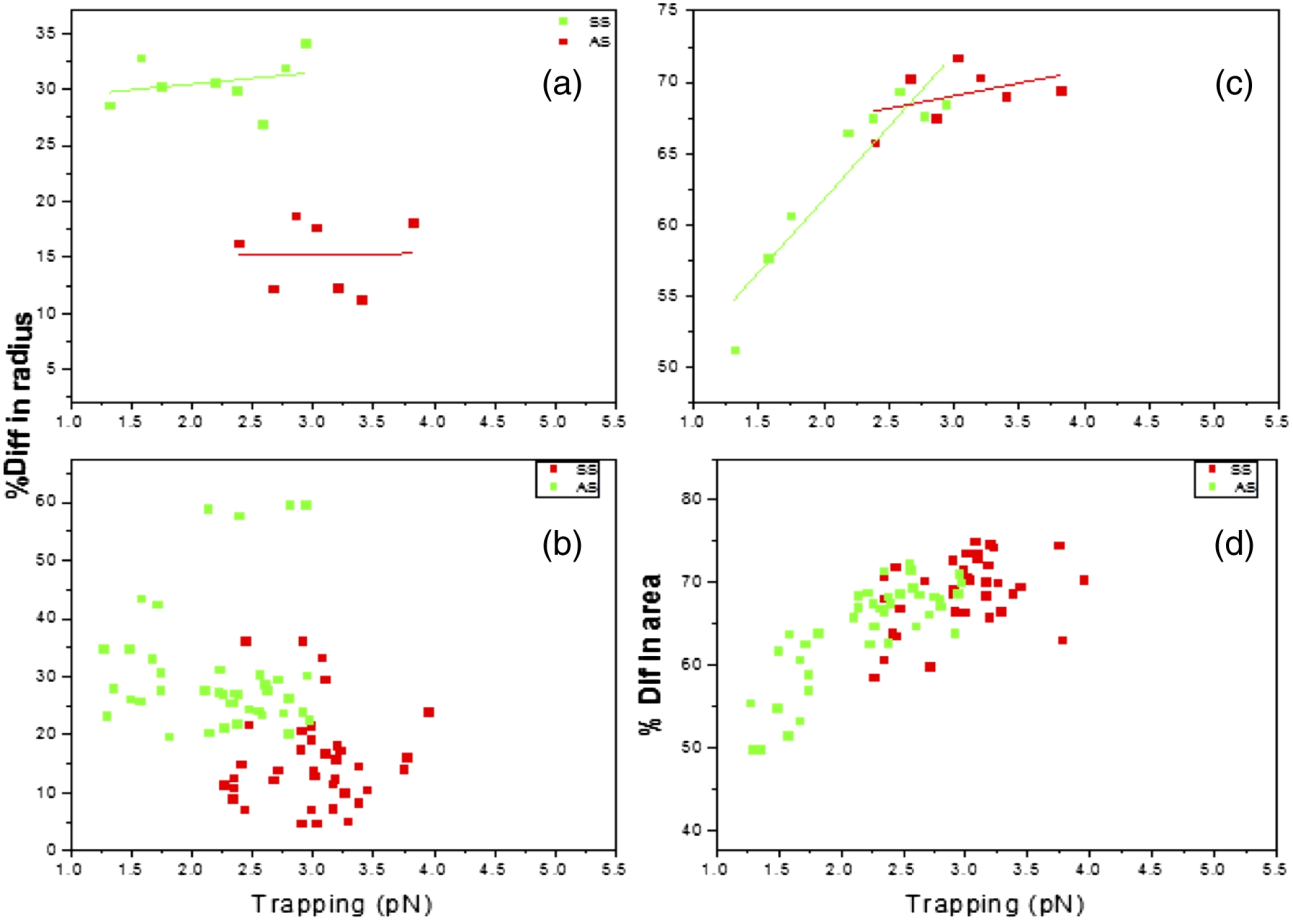
(a), (b) Radius percent difference versus trap; (c), (d) area percent difference versus trapping force HbAS (green) and HbSS (red).

Figure 6 displays the change of radius and area of the trapped cell relative to the radius and area of the free cell for HbAS (green) and HbSS (red). Figures 6(a) and 6(b) show the change in radius for HbAS (green) and HbSS (red) samples; Figs. 6(c) and 6(d) display the change in area of trapped and free cells for HbAS (green) and HbSS (red). The reduced data for HbSS shown in Figs. 6(b) and 6(d) were obtained first by sorting by the trapping force and deleting two minima (points on the far left) and one maximum (points on the far right). This is then followed by sorting by radius and remove two maxima from the top and two minima from the bottom side. Finally, we sorted by area percentage difference and removed three maxima and one minimum. For HbAS, we sorted by trapping force and deleted two maxima from the top and two minima the bottom. Next sorting by radius and removed two from the maxima and the minimum follow sorting by area and eliminated three maxima from the top only. The result displayed in Fig. 6(a) for HbAS shows a larger radius percent difference than HbSS. Since HbAS had a higher percentage difference in area for the smaller force, then HbAS cells stretched more than HbSS cells. Additionally, the HbSS cell has a larger area percent difference at the higher force. HbAS under trapping force stretched faster than HbSS. The radius percent difference in Fig. 5(a) clearly shows that HbAS has a higher elastic property than HbSS. HbAS can be deformed with less force than HbSS since the hydrophobic lipid region of sickle cells’ membranes is less fluid.^55^

**Fig. 7:**
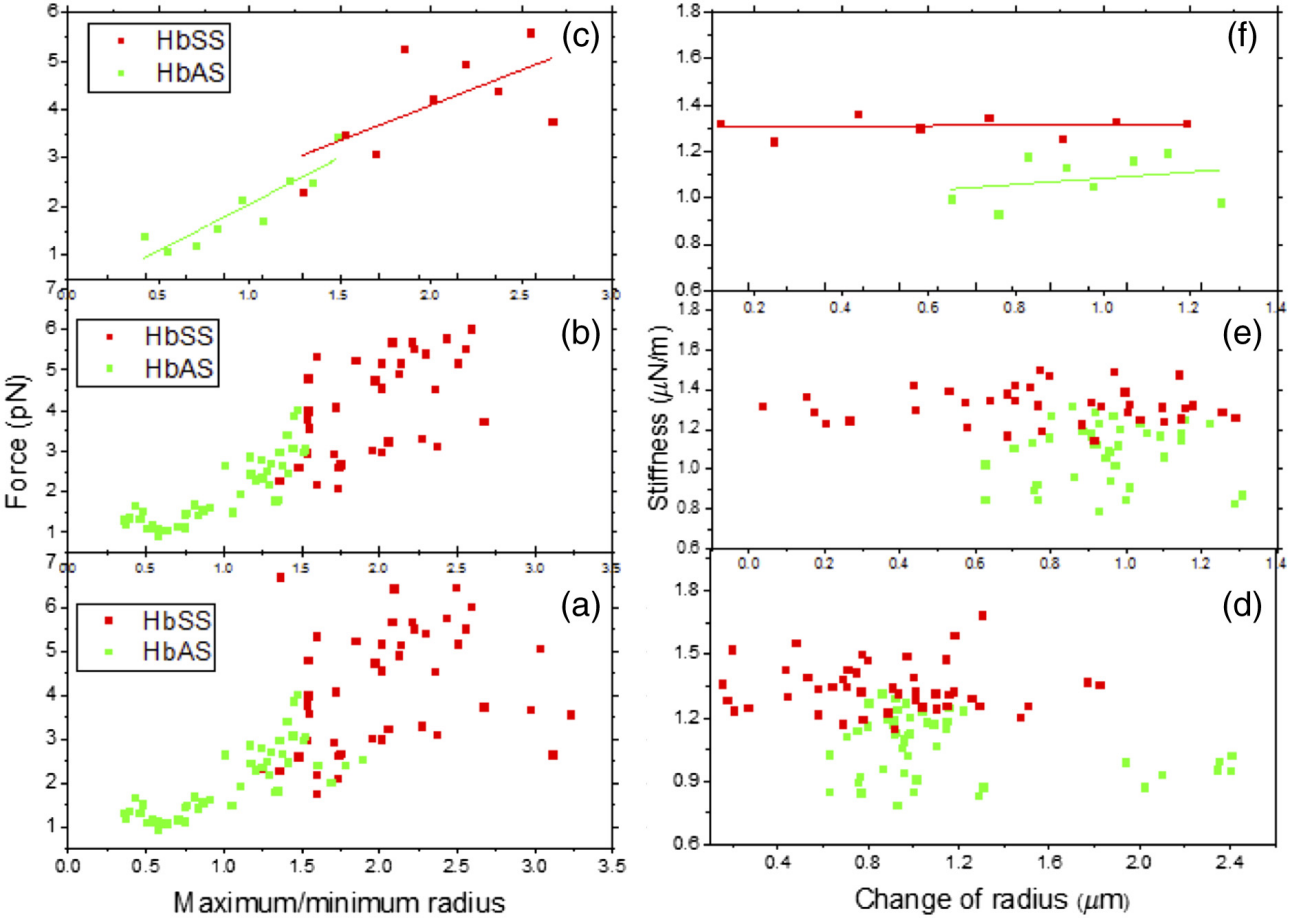
(a)–(f) The fraction of max radius over min radius and stiffness versus radius for the HbAS (green) and HbSS (red) RBCs.

Figures 7(a)–7(c) show the ratio of maximum over the minimum radius versus trapping force, and Figs. 7(d)–7(f) are stiffness versus radius. In Figs. 7(a) and 7(d), the whole data of HbSS (red) and HbAS (green) RBC samples are displayed. The data displayed in Fig. 7(b) were reduced from (a) HbSS by eliminating one minimum and three maxima after sorting by the ratio of maximum/minimum and four maxima sorting by the dragged force and five HbAS maxima by sorting by the dragged force. Figure 7(e) reduced data comes from Fig. 7(d) by deleting four HbSS maxima and then sorting in terms of stiffness and radius change, respectively. Further, eight HbAS maxima were deleted and sorted by change in radius. Finally, Figs. 7(c) and 7(f) are displayed using the data reduction method and the result in Fig. 7(c) shows that HbAS is higher in maximum to minimum ratio than HbSS, however, Fig. 7(f) confirms that HbSS (red) is stiffer than HbAS (blue). The results for the ratio of maximum to minimum radius also show the same behavior in the elasticity of the RBCs in the two blood samples. This alteration of rigidity was due to the lipid compositions which depends on the cholesterol concentration that results in less fluid on the HbSS.

### 3.2 Trapped Compared to Stretched for SCA and SCT Cells

The relative change of stretched radius and area from the trapped cells can be calculated from and $\Delta A=A_{ò}-A_{O}$, respectively. The percent difference for both SCT and SCA has been calculated as shown in Eqs. (18) and (19), respectively, where $R=R_{ò}$ and $A=A_{ò}$.

**Fig. 8:**
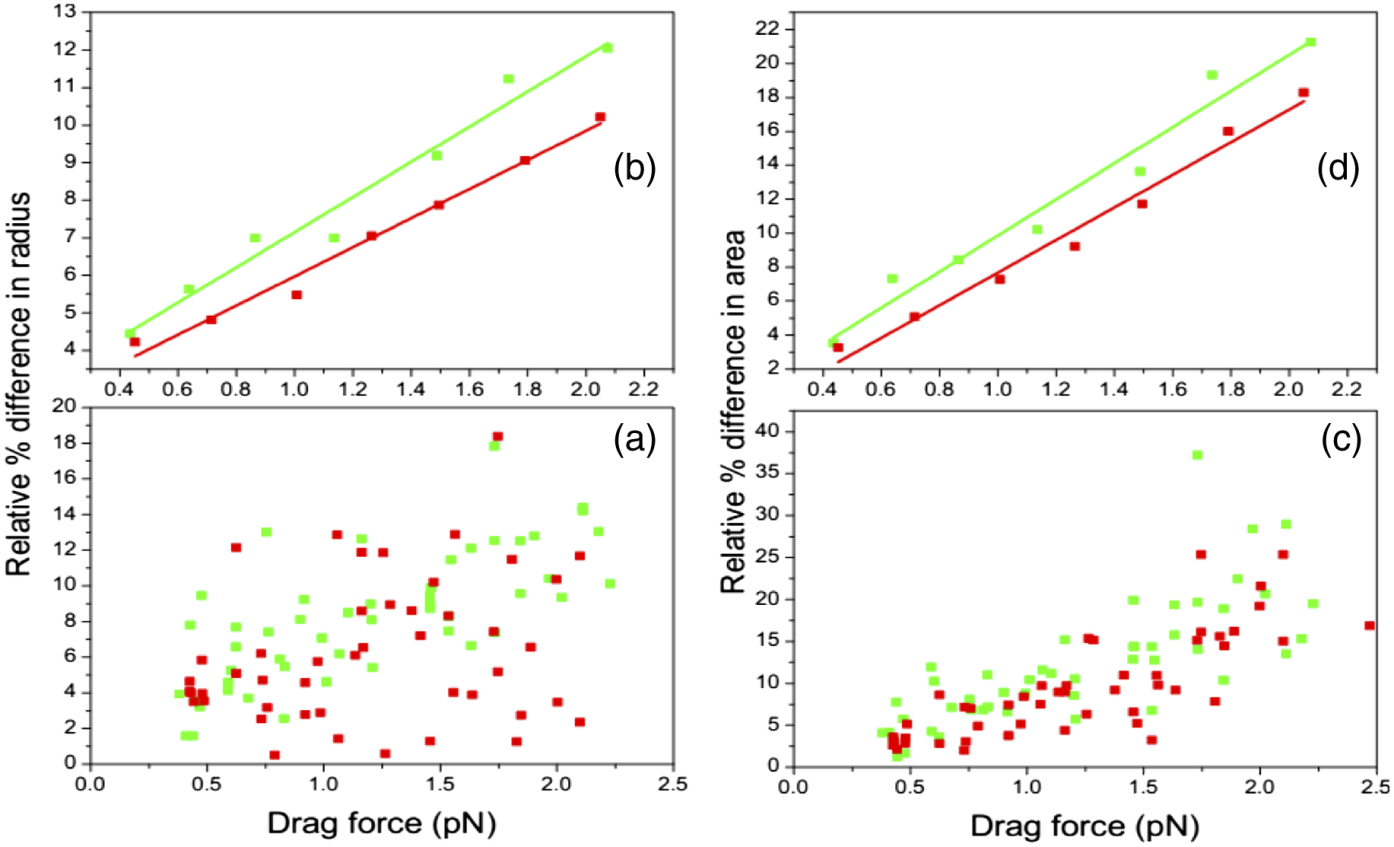
(a)–(d) The relative percent difference versus drug force for HbAS (green) and HbSS (red).

Figures 8(a) and 8(c) display 49 HbAS and 49 HbSS samples. Each HbAS and HbSS are divided in seven groups and exposed with seven different velocities. Figures 8(b) and 8(d) show the averaged data from the seven groups of relative percentage difference in radius and area versus drag force for HbAS and HbSS data, respectively. The results confirm that the relative change of HbAS has higher value of relative percent difference than the HbSS RBCs.

The results for t-test statistics for the radius percentage difference and stiffness are given in Fig. 5. These results show that there was a significant difference between the HbAS and HbSS groups. At 0.05 significant level, the percentage difference in radius (free from trapped) and (trapped from stretched) in the HbAS and HbSS. We ran a two-sample t-test with both equal and not equal variance assumptions: the mean values for both (free from trapped) radiuses and (trapped from stretched) radiuses were found to be statistically significantly different at the 0.05 level. The stiffness of the HbAS and HbSS were also statistically significantly different at 0.01 level. Based on these results, we can conclude that the mean relative percentage difference for HbAS is significantly greater than that of HbSS, and the mean stiffness for HbAS is significantly smaller than HbSS. Therefore, t-test statistics analyses further confirm that HbAS has more elastic properties than HbSS.

It is well demonstrated in the literature that the major factors affecting cells’ higher stiffness is lipid composition.^56^^–^^60^ Bilayer biomechanical and structural features depend on the lipid composition, independent of the molecular models, and both the galactosylceramide and sphingomyelin lipids increase the order of aliphatic tails and resistance to water penetration.^55^ This alters the fluidity of the hydrophobic lipid region of sickle cell membranes which become less fluid compared with those of normal erythrocytes due to the hydrophobic lipid region in sickle cell membranes being more rigid.^56^ It has been shown that the bilayers’ stiffness can be augmented by about 30% of galactosylceramide. Growth of bilayer width has been associated with sugar–sugar interactions of galactosylceramide bonded lipids and hydrogen-bond phosphocholine.^52^ Thus informing us on lipids behavior in membranes at the molecular level, we can argue that the alteration of rigidity is a result to the lipid compositions.^57^^,^^58^ This composition depends on the cholesterol concentration which results in less fluid on the HbSS. Another factor may also be the negative potential at the surface of the sickle cell membrane as it is decreased.^55^

## 4 Comparison to Prior Results from Optical Trapping Studies of RBCs

In our study, the trapping force for the deformation of the HbAS was found to be 0.26 pN and for HbSS 0.33 pN. We report here that the stretching drag force of HbAS is 1.2 pN and for HbSS 1.3 pN. The stiffness of the HbSS has a higher value (4.47  μN/m) than that of HbAS (1.08  μN/m). In a recent study, the force was calculated using a spherical model and the elastic shear modulus was reported as 2.5±0.4  μN/m.^61^ This was deduced from the slope of the linear relationship between the membrane equatorial deformation and the applied force. A much earlier study using the same model reported a large deformation from the stretching force in excess of 400 pN, which is large compared to our result. However, the force in the range of 20 to 30 pN shows small deformation.^62^ Additionally, the membrane shear modulus values are found to be in the range of 11 to 18  N/m, at average strains, which is typical of large deformation. Also the initial shear modulus values were in the range of 19 to 30  N/m, which are much larger shear modulus values than we found. It was demonstrated that the elasticity of RBC measured similar to us: for HbAS $(0.132-8.553)\times 10^{-3}\;\mathrm{dyn}/\mathrm{cm}$ and for HbSS $(0.124-15.542)\times 10^{-3}\;\mathrm{dyn}/\mathrm{cm}$.^63^

Solomon et al.^64^ showed that the relative changes in longitudinal (maximum) and the mean diameters appear to be higher for SCA than SCT when the cells are trapped or trapped and dragged by different speeds with respect to the corresponding diameters of the free cells. Byun et al.^65^ used optical tweezers and micropipette aspiration techniques to study the mechanical deformation of the RBC and reported shear modulus of sickle RBCs in the range of 8 to $20\;\mu N\;m^{-1}$. Further, AFM measurements have found that the Young’s modulus of SCA RBCs is stiffer than normal RBCs with a widely distributed Young’s modulus ranging from 3 to 50 kPa.^66^ All these results are in agreement with our results described in the first paragraph. Unlike most techniques that are limited force calibration, this work depends on the measurement of the refractive index of the cell, medium, and spot size.

## 5 Limitations

This study has two limitations in the experimental technique and theoretical model that we could improve for better and more precise predictive results. In the experimental technique, we used one trap to deform a single cell, which creates a limitation in the number of cells available for us to study. The more cells are studied, the better predicting the RBCs’ elastic properties in the specific blood sample. We can improve this limitation by creating multiple traps using an acoustic-optical deflector.^67^^–^^69^ Such a device allows trapping multiple cells at a time, and we can increase the number of cells studied per blood sample. The second limitation is in the theoretical model. In the theoretical model, we modeled the shape of the RBCs with a thin cylinder. However, the actual shape for normal RBCs is more of a donut shape, thinner in the middle, and thicker outside. A model that considers this variation in the thickness of the RBCs can better predict the trapping force.

## 6 Conclusion

By developing a theoretical model for different trapping forces, we have also studied the elasticity of RBCs in SCT (with hemoglobin type HbAS) and in SCA (with hemoglobin type HbSS). We compared cells’ elasticity property using radius and area relative percent difference and stiffness versus trapping force. The validity of these results was also investigated using descriptive statistics. The sizes of the RBCs in SCA were found to be larger than those in SCT. The results for the quantities describing the elasticity of the cells had consistently showed that the RBCs in the SCT display lower rigidity and higher deformability than the RBCs with SCA. This behavior, in particular, is distinctly shown by the stiffness constant versus size (measured by the radius) results. This property in elasticity is also shown in the results for the relative change of the percent difference of individual RBCs in SCT (HbAS) and SCA (HbSS).
